# Supplementary material for: Gut microbiota-associated non-cholesterol sterol dysregulation modulates immune reconstitution during antiretroviral therapy in people living with HIV
Source: Microbiol Spectr. 2025 Jul 24;13(9):e01404-25. doi: 10.1128/spectrum.01404-25 (PMC12403673; doi:10.1128/spectrum.01404-25)
Supplement: Supplemental figures and tables — Fig. S1 to S8, and Tables S1 and S2. [file spectrum.01404-25-s0001.docx]

Additional file for

**Gut microbiota-associated non-cholesterol sterol dysregulation modulates immune reconstitution during antiretroviral therapy in people living with HIV**


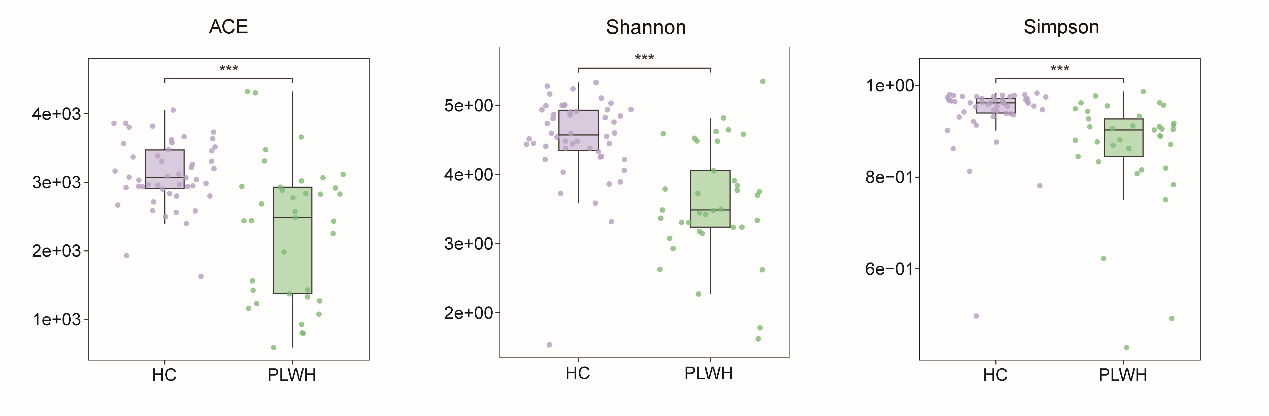


**Figure S1.** Comparison of alpha diversity between people living with HIV (PLWH, *n* = 37) and healthy controls (HC, *n* = 50), presented as median ± interquartile range (IQR); ****P* < 0.005 by Mann–Whitney *U* test.


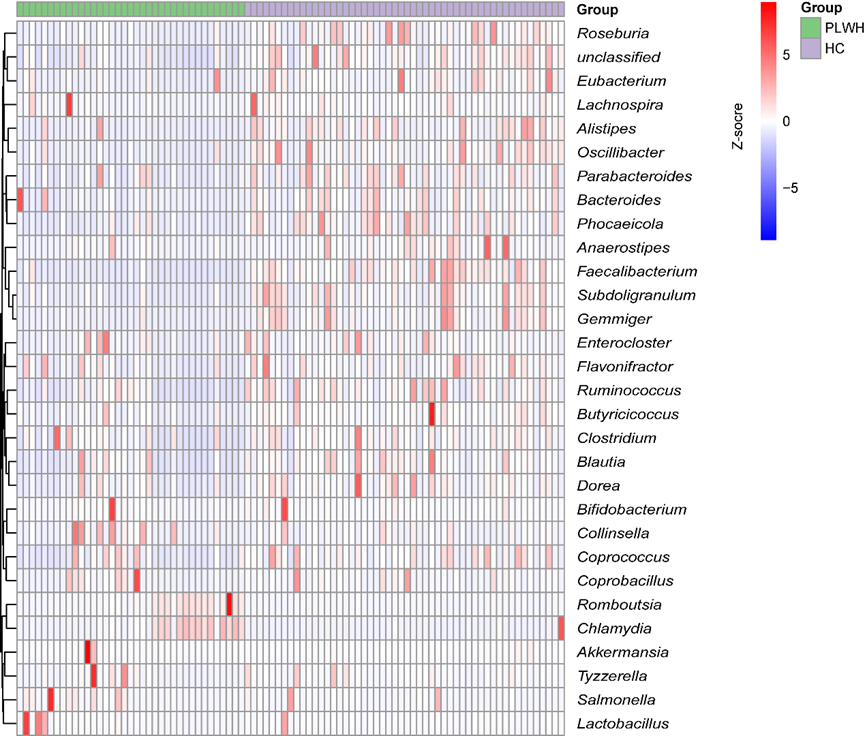


**Figure S2.** Heatmap of the top 30 differentially abundant genera (log2 fold-change > 2, FDR < 0.05) between PLWH (*n* = 37) and HC (*n* = 50). Species relative abundance was normalized by Z-score (row-wise).


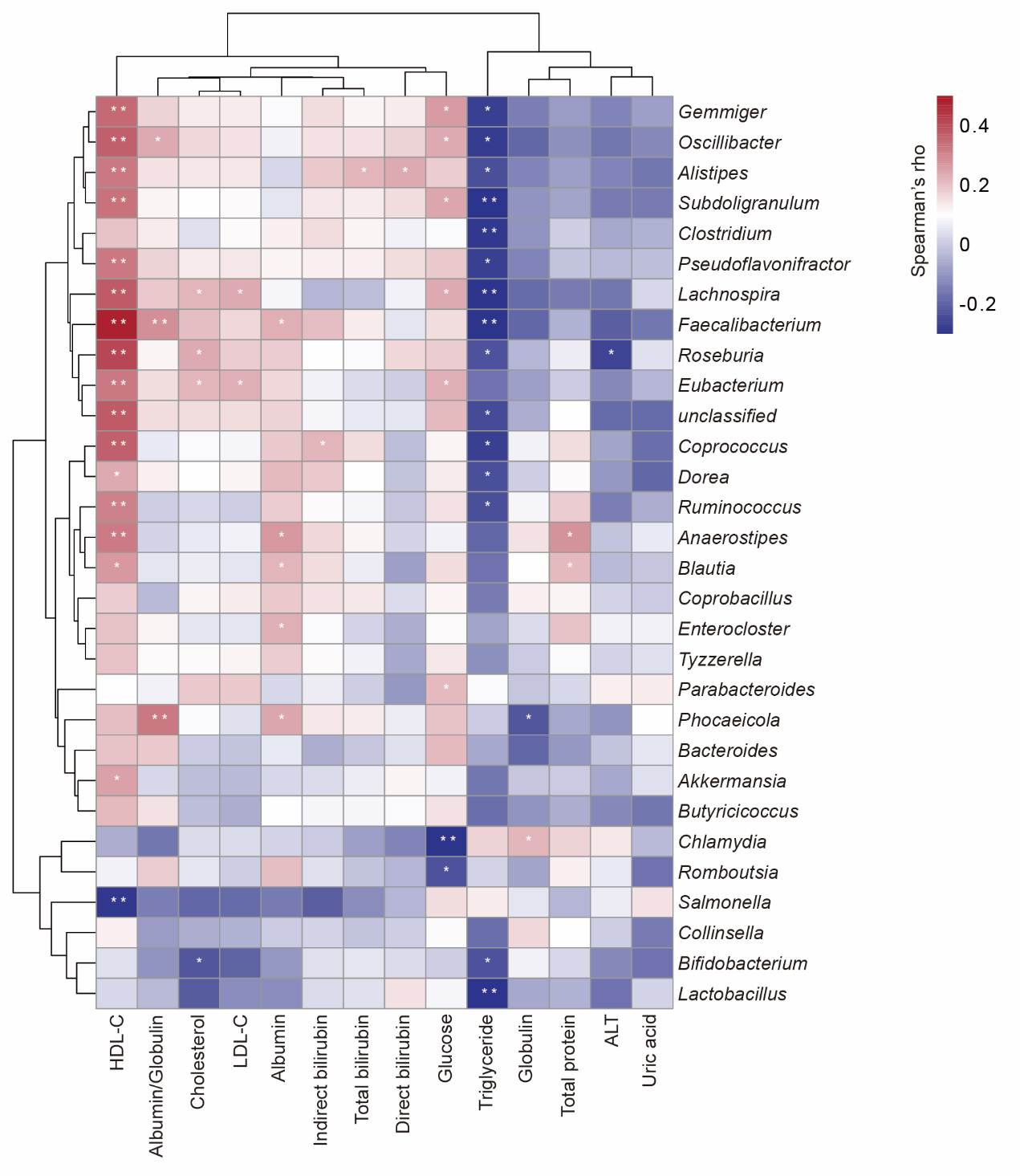


**Figure S3.** Spearman’s correlation heatmap showing associations between the top 30 differential genera (identified between PLWH and HC) and host clinical parameters in participants (*n* = 87). Color intensity reflects Spearman’s *ρ* (*q*-value < 0.2, Benjamini–Hochberg corrected); asterisks denote significant correlations (**P* < 0.05, ***P* < 0.01). ALT, alanine aminotransferase; LDL-C, low-density lipoprotein cholesterol; HDL-C, high-density lipoprotein cholesterol.


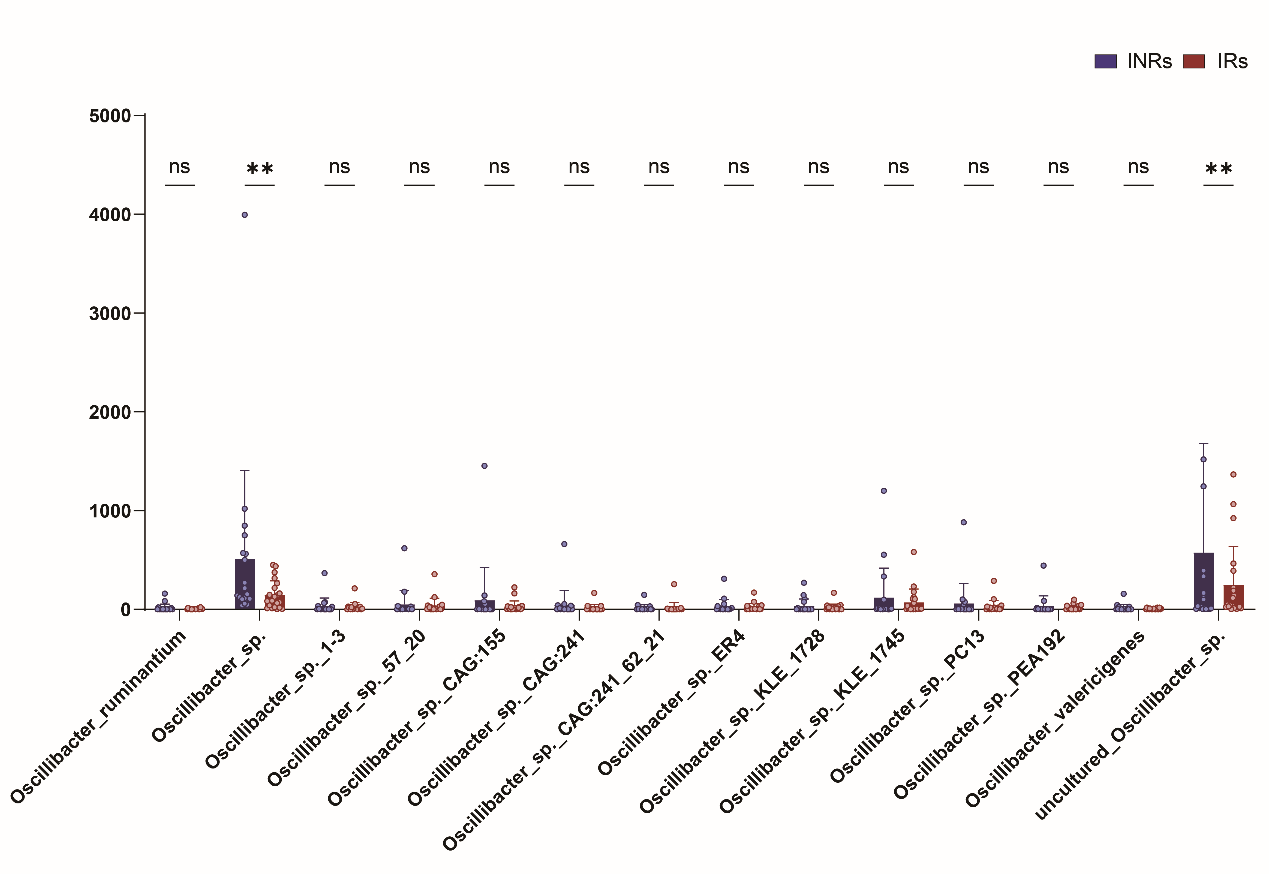

**Figure S4.** Comparative abundance of species from *Oscillibacter* genus between the immunological non-responders (INRs, *n* = 24) and immunological non-responders (IRs, *n* = 13) groups (median ± IQR; ***P* < 0.01 by Mann–Whitney *U* test).


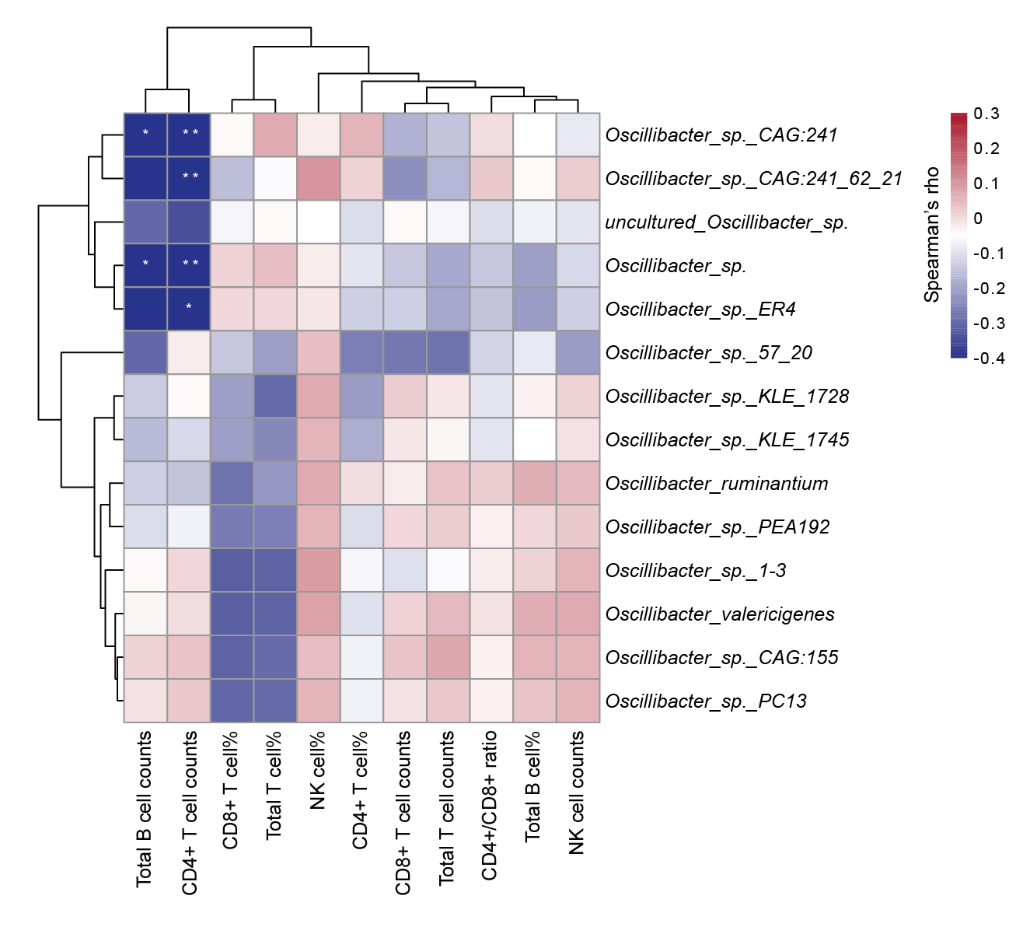


**Figure S5.** Spearman’s correlation heatmap between serum non-cholesterol sterols and cholesterol levels and immune cells subset in PLWH (*n* = 37). Color intensity reflects Spearman’s *ρ* (*q*-value < 0.2, Benjamini–Hochberg corrected); asterisks denote significant correlations (**P* < 0.05, ***P* < 0.01).


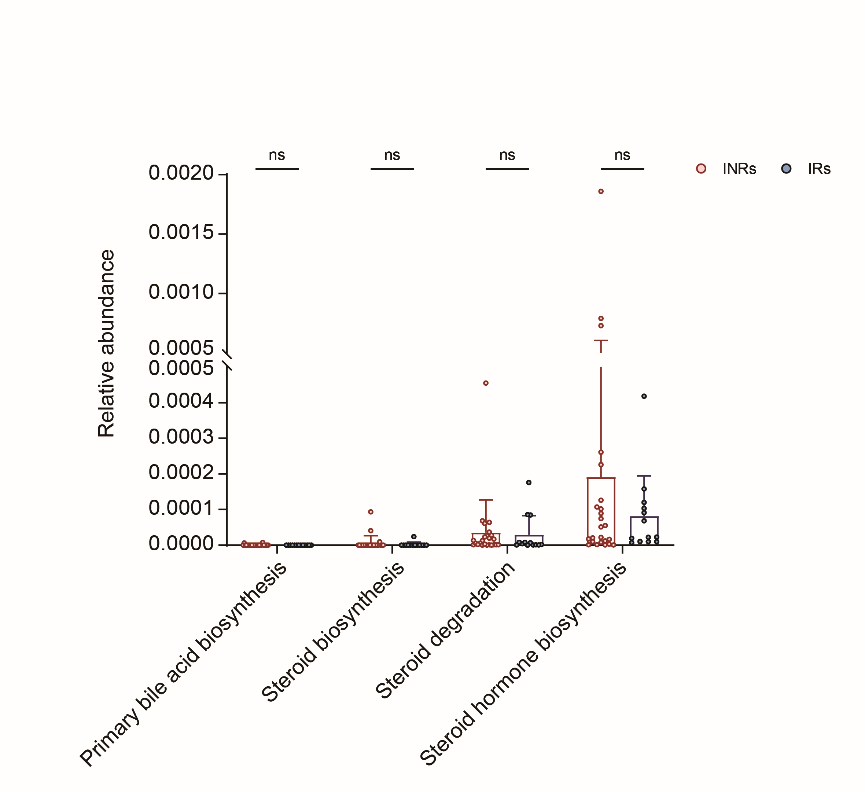


**Figure S6.** Relative abundance of KEGG pathways (level 3) between the INRs (*n* = 24) and IRs (*n* = 13) groups (ns, not significant).


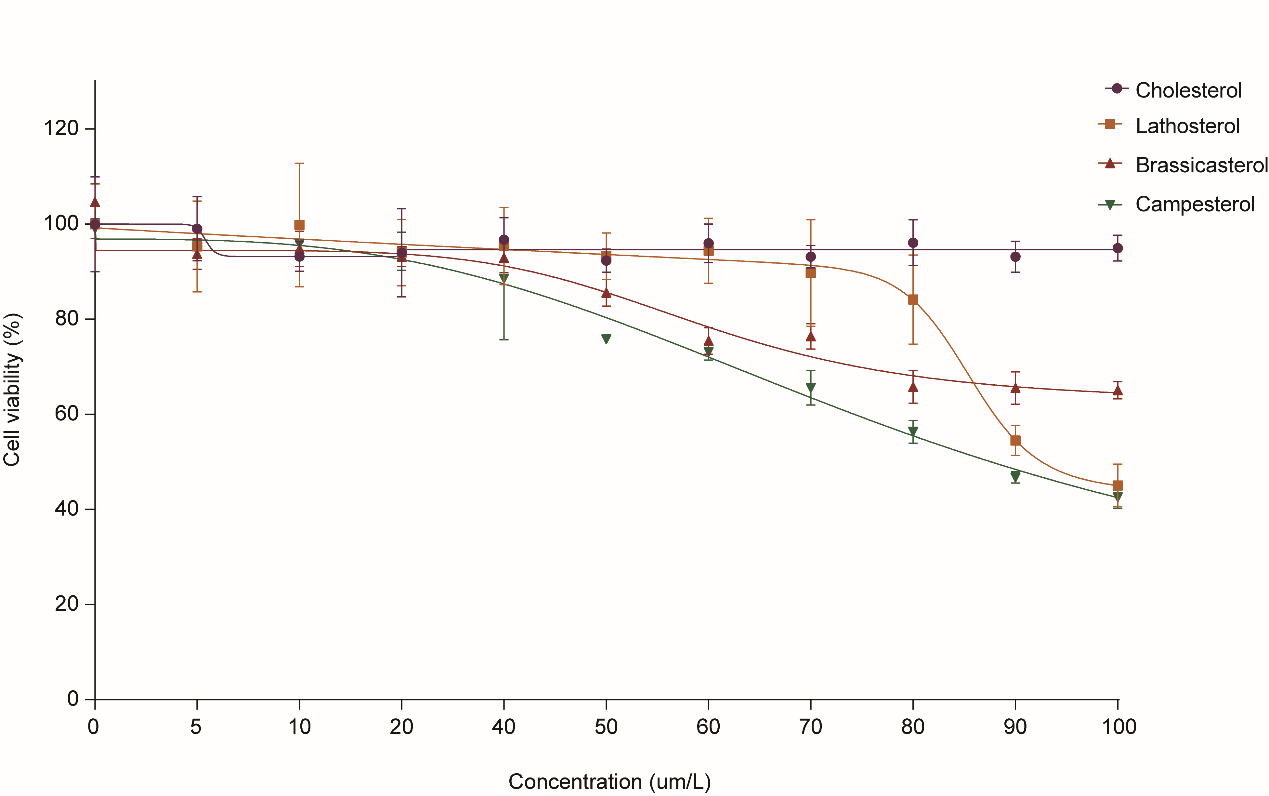


**Figure S7.** Effect of metabolites on the cell viability of PBMCs.


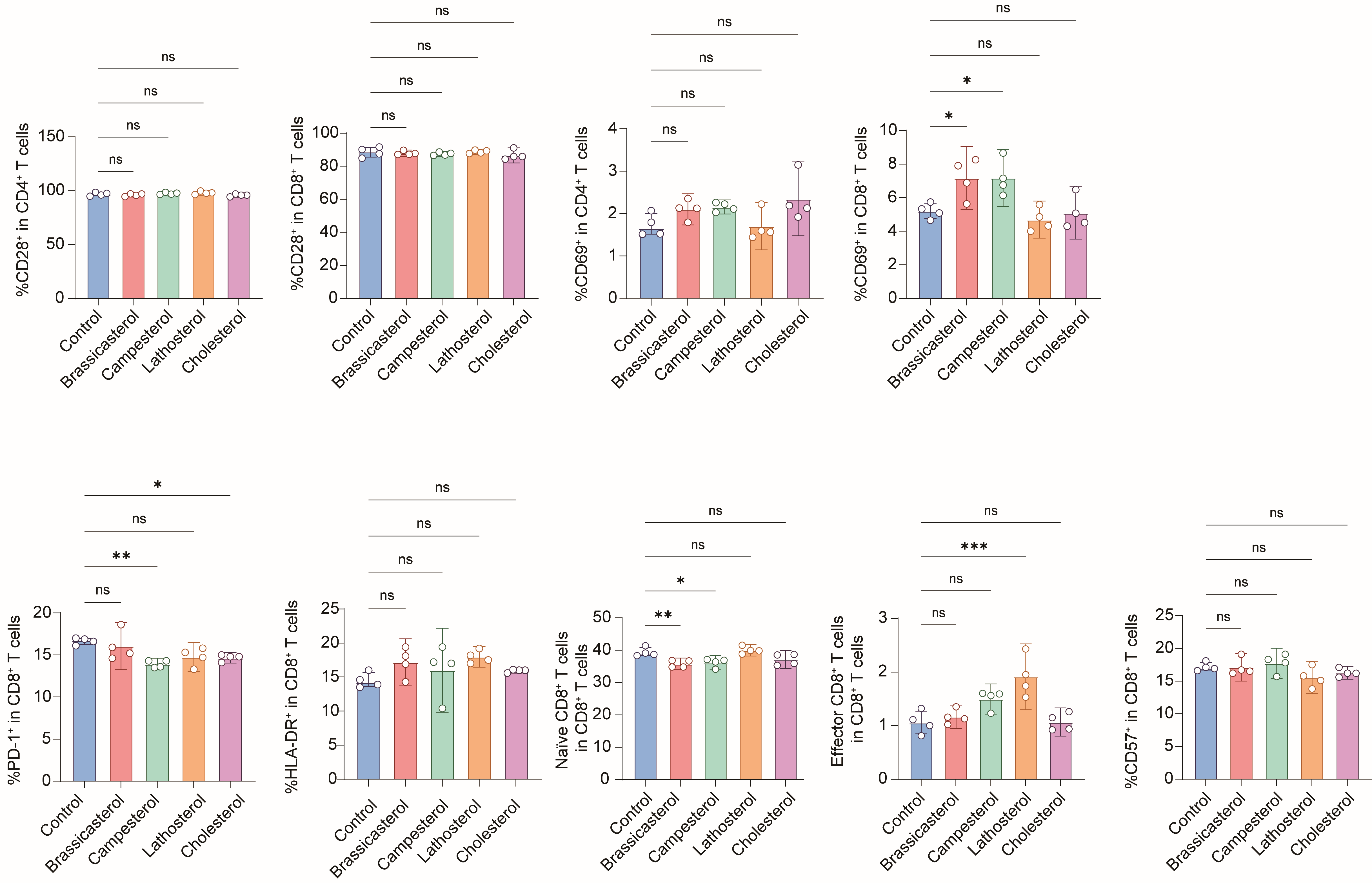


**Figure S8.** Flow cytometry quantification of T cell markers following 48-h stimulation with 40 μM of brassicasterol, campesterol, lathosterol, or cholesterol. (*n* = 4 donors; mean ± SEM; **P* < 0.05, ***P* < 0.01, ****P* < 0.005 by one-way ANOVA with Tukey’s post-hoc test). Related to Figure 4.

**Table S1.** Clinical characteristics of healthy controls and people living with HIV.

|  | HC (*n* = 50) | PLWH (*n* = 37) | *P*-value |
| --- | --- | --- | --- |
| Age (year) | 36(30–45) | 39(35–45) | 0.286 |
| Sex |  |  | 0.000 |
| Female | 12(24%) | 1(2.70%) |  |
| Male | 38(86%) | 36(97.30%) |  |
| Total bilirubin (μmol/L) | 9.2(8.2–12.4) | 8(5.8–9.9) | 0.007 |
| Direct bilirubin (μmol/L) | 3.2(2.7–3.8) | 3.1(2.15–3.9) | 0.286 |
| Indirect bilirubin (μmol/L) | 6.2(4.9–8.4) | 4.8(3.7–6.25) | 0.004 |
| ALT (U/L) | 20(14–30) | 23(16.5–47.5) | 0.089 |
| Total protein (g/L) | 73.9(71.3–76.5) | 74(71.15–79.1) | 0.989 |
| Albumin (g/L) | 47.2(46–49) | 44.1(41.4–47.95) | 0.003 |
| Globulin (g/L) | 26.8(23.9–29.8) | 29(26.1–32) | 0.042 |
| Albumin/globulin | 1.8(1.6–2) | 1.5(1.4–1.75) | 0.000 |
| Uric acid (μmol/L) | 338(254–392) | 345(286.5–389) | 0.634 |
| Glucose (mmol/L) | 4.65(4.3–5.11) | 4.29(3.99–4.705) | 0.013 |
| Triglyceride (mmol/L) | 1.26(0.86–2.04) | 1.58(1–2.175) | 0.027 |
| Cholesterol (mmol/L) | 4.79(4.21–5.75) | 4.69 (4.265–4.995) | 0.562 |
| HDL-C (mmol/L) | 1.33(1.02–1.61) | 0.92(0.73–1.18) | 0.000 |
| LDL-C (mmol/L) | 2.55(2.12–3.48) | 2.13(1.725–3.185) | 0.055 |
| ART months | - | 79(50-111) |  |
| HIV RNA copies | - | ND |  |
| Current CD4 + T cell counts (/μL) | - | 345.3(200-455.5) |  |
| Current CD8 + T cell counts (/μL) | - | 655.25(508.2-854.9) |  |
| Diagnosis-to-treatment interval (month) | - | 15(4-39) |  |

Data are presented in median (IQR, interquartile range) and number (percentage, %). Fisher exact test was used to calculate the *P*-value of categorical variable. Mann–Whitney *U* test was used to calculate the *P*-value of continuous variables. *P* < 0.05 is considered to be statistically significant. PLWH, people living with HIV; ALT, alanine aminotransferase; LDL-C, low-density lipoprotein cholesterol; HDL-C, high-density lipoprotein cholesterol; ND, not detected; ART, antiretroviral therapy.

**Table S2.** Clinical characteristics of INRs and IRs.

|  | INRs (*n* = 24) | IRs (*n* = 13) | *P*-value |
| --- | --- | --- | --- |
| Age (year) | 37(33.5-42) | 41(35.5-45.5) | 0.625 |
| Sex |  |  | 0.649 |
| Female | 1(4.17%) | 0(0%) |  |
| Male | 23(95.83%) | 13(100%) |  |
| ART months | 61(50.5-78) | 80(43-112) | 0.632 |
| HIV RNA copies | ND | ND | - |
| NK cell counts (/μL) | 125(70-264.7) | 349.7(200.4-478.4) | 0.003 |
| NK cell (%) | 11.9(5.84-19.75) | 14.5(12.35-19.29) | 0.202 |
| Total T cell counts(/μL) | 1084.7(734-1243.3) | 1350(1142.6-1686.8) | 0.001 |
| Total T cell (%) | 73.9(68.4-87.3) | 71.8(65.05-75.5) | 0.296 |
| Total B cell counts (/μL) | 113.3(42-149.85) | 200.4(129.65-250) | 0.015 |
| Total B cell (%) | 7.1(4.75-12.4) | 10.2(7.7-11.5) | 0.344 |
| Current CD4+/CD8+ ratio | 0.45(0.355-0.555) | 0.86(0.595-1.04) | 0.003 |
| Current CD4+ T cell counts (/μL) | 288.6(192.65-345.3) | 487.9(452.25-682.2) | 0.000 |
| Current CD4+ T cell (%) | 20.2(17.5-22.35) | 30.9(24.65-34.8) | 0.002 |
| Current CD8+ T cell counts (/μL) | 642.4(460.35-760.45) | 721.7(566.05-832.15) | 0.070 |
| Current CD8 + T cell (%) | 49.2(39.7-60.205) | 38.2(30-40.7) | 0.008 |
| Diagnosis-to-treatment interval (month) | 12(6-30) | 16(2-40) | 0.423 |
| Total bilirubin (μmol/L) | 7(5.65-7.9) | 9.1(6.05-10) | 0.048 |
| Direct bilirubin (μmol/L) | 2.5(2-3.2) | 3.1(2.15-3.8) | 0.157 |
| Indirect bilirubin (μmol/L) | 4.1(3.05-4.8) | 5.8(4-6.65) | 0.074 |
| ALT (U/L) | 33(19.5-53) | 23(13.5-46.5) | 0.582 |
| Total protein (g/L) | 73.7(71.25-76.05) | 74.4(72-80.8) | 0.511 |
| Albumin (g/L) | 44.1(42.85-48.25) | 45.4(41.4-47.65) | 0.292 |
| Globulin (g/L) | 28.8(26.75-29.55) | 31.5(25.95-32.6) | 0.423 |
| Albumin/globulin | 1.5(1.4-1.85) | 1.5(1.25-1.65) | 0.276 |
| Uric acid (μmol/L) | 331(302-362.5) | 347(246-389) | 0.790 |
| Glucose (mmol/L) | 4.3(4.015-4.455) | 4.2(3.94-4.705) | 0.873 |
| Triglyceride (mmol/L) | 1.5(1-1.85) | 1.73(1.115-2.33) | 0.606 |
| Cholesterol (mmol/L) | 3.9(3.58-4.79) | 4.13(3.195-4.655) | 0.606 |
| HDL-C (mmol/L) | 0.92(0.8-1.145) | 0.97(0.74-1.18) | 0.582 |
| LDL-C (mmol/L) | 2.13(1.765-2.78) | 2.1(1.615-2.65) | 0.736 |

Data are presented in median (IQR, interquartile range) and number (percentage, %). Fisher exact test was used to calculate the *P*-value of categorical variable. Mann–Whitney *U* test was used to calculate the *P*-value of continuous variables. *P* < 0.05 is considered to be statistically significant. INRs, Immunological responders; IRs, Immunological non-responders; ART, antiretroviral therapy; ND, not detected; ALT, alanine aminotransferase; LDL-C, low-density lipoprotein cholesterol; HDL-C, high-density lipoprotein cholesterol.
